# Clinical features and demographic characteristics of gestational trophoblastic neoplasia: Single center experience and the SEER database

**DOI:** 10.17305/bb.2023.9092

**Published:** 2024-02-01

**Authors:** Yue-min Hou, Pei-pei Li, Hui Yu, Fang Feng, Xin-yi He, Bi-han Chen, Jia-ling Li, Hao-yan Yao, Rui-fang An

**Affiliations:** 1 Department of Gynecology and Obstetrics, The First Affiliated Hospital of Xi’an Jiaotong University, Xi’an, Shaanxi, China

**Keywords:** Gestational trophoblastic neoplasia (GTN), clinical features, demographic characteristics, prognosis, SEER, survival analysis

## Abstract

The aim of this study was to analyze the clinical features and demographic characteristics of gestational trophoblastic neoplasia (GTN) patients, specifically choriocarcinoma (CC), placental site trophoblastic tumor (PSTT), and epithelioid trophoblastic tumor (ETT). We utilized data from a local hospital and the Surveillance, Epidemiology, and End Results database (SEER), as well as survival outcomes of CC in the SEER database. Additionally, we used multiple risk factors to create a prognostic nomogram model for CC patients. The study included GTN patients from the SEER database between 1975 and 2016 as well as those from the First Affiliated Hospital of Xi’an Jiaotong University between January 2005 and May 2022. Related factors of patients were compared using the chi-square (χ^2^) or Fisher’s exact test. For assessing overall survival, we employed the Kaplan–Meier method and log-rank test. To construct the nomogram, we used Cox regression. Statistically significant differences were found between CC and PSTT/ETT patients in terms of surgery in local hospital, as well as age and year of diagnosis in the SEER database. Moreover, significant differences were observed between low and high (HR)/ultra-high risk (UHR) groups regarding FIGO stage, surgery, and chief complaint at the local hospital, and FIGO stage, surgery, and unemployment in the SEER database. The Cox regression analysis confirmed that age, race, surgery, marital status, FIGO stage, and unemployment were correlated with CC prognosis. Furthermore, the analysis showed that patients aged 40 years or older and those with FIGO stage III/IV were independent prognostic factors of CC. The study indicates that atypical symptoms or signs may be the main reasons for HR/UHR patients to seek medical treatment. Therefore, providing multidisciplinary care is recommended for CC patients experiencing psychological distress due to unfavorable marital status or unemployment.

## Introduction

Gestational trophoblastic neoplasia (GTN) is a relatively rare malignancy arising from the placenta. It frequently occurs after pregnancy and is more common after pregnancy with hydatidiform mole [1–3]. It consists of invasive mole, choriocarcinoma (CC), placental site trophoblastic tumor (PSTT), and epithelioid trophoblastic tumor (ETT) [1, 4]. Invasive mole has a lower grade of malignancy and a better prognosis. The incidence of CC is low (1–9/40,000 pregnancies), while PSTT and ETT are even less common [1]. The lesion site of GTN is mainly in the uterus, but it can also involve surrounding tissues, such as the fallopian tubes and ovaries [4]. It can metastasize to the lungs, vagina, brain, and liver [1, 4, 5]. The clinical features of GTN depend on the type of disease, the lesion site, and the patient’s general condition before the disease [4, 6]. Abnormal vaginal bleeding is the most common symptom because trophoblastic tumor has fragile vessels [7]. Distant metastases of GTN may present with cough, hemoptysis, chest and abdominal pain, headache, and other symptoms [8]. Chemotherapy is the main treatment for GTN, and surgery may be an adjuvant procedure [1, 9, 10]. 

The FIGO/WHO defined several prognostic risk factors for CC [1, 11]. FIGO scores ranged from 0 to 6 for low risk, ≥ 7 for HR, and ≥13 for ultra-high risk UHR [11–13]. Limited information has been available on the UHR subgroup because it is rare. Methotrexate (MTX) or actinomycin-D (Act-D) can be used to treat low-risk GTN. Patients with resistance to monotherapy are treated with multi-agent chemotherapy [14]. Non-metastatic, low-risk GTN patients who do not require fertility preservation may also opt for hysterectomy [3]. Approximately 1/4 of patients develop resistance or toxic reactions to initial single-agent chemotherapy [11]. Up to 70%–80% of patients with a score of 5 or 6 have the highest rate of resistance to initial single-agent chemotherapy [3]. Therefore, the current question is whether GTN patients should be reclassified without compromising treatment efficacy, to minimize chemotherapy resistance and toxicity. Others have proposed to determine new prognostic risk factors to redefine the FIGO/WHO scoring system [4].

PSTT and ETT consist of intermediate trophoblastic cells in the placental region and share several common features [15]. Both types of tumors grow very slowly and can occur after several types of previous pregnancies [4, 16]. They produce very little beta-human chorionic gonadotropin (β-hCG) and metastasize at later stages [4, 17]. In general, both tumors are less sensitive to chemotherapy than CC. There are three independent poor prognostic factors for PSTT and ETT: mitotic index of tumor cells >5–10 high power fields (HPF), two or more years after last pregnancy, and FIGO stage IV. Currently, there are no uniform guidelines for the treatment of patients with PSTT and ETT. The primary treatment for most patients with PSTT or ETT consists of hysterectomy and resection of all suspicious pelvic and retroperitoneal lymph nodes. And HR patients with PSTT or ETT should receive multi-agent chemotherapy on this basis. When there are no metastases, the disease survival rate is more than 90%, and when there are metastases, it drops to 50%–60% [4, 18]. 

Some studies suggest that patients with GTN usually suffer from various psychological complaints, such as distress, depression, and anxiety [19, 20]. Because GTN is a rare neoplasm, there are few studies that address these risk factors, and many of them involve only a small number of cases. The SEER database is a cancer database that captures approximately 30% of the population data in the United States. It contains several data that indirectly reflect the psychosocial outcomes, such as marital status, education, income, unemployment, and smoking habits. In this study, we extracted data on patients diagnosed with GTN between 1975 and 2016 from the SEER database to examine clinical features, demographics, and survival data. We also collected and reviewed the medical records of patients with a histopathological diagnosis of GTN at the First Affiliated Hospital of Xi’an Jiaotong University between January 2005 and May 2022. It is known that no previous study has combined clinical and demographic characteristics to predict the survival probability of patients with CC, which limited the identification of risk factors for death occurrence. Therefore, we investigated this question. Nomograms can quantify prognostic risk factors by using line segments with scales and predict the risk of disease occurrence more intuitively and individually. It is known that there is no nomogram that combines psychosocial factors specifically to predict the survival rate of CC patients. Therefore, we created a nomogram for CC patients using the SEER database to evaluate the 5-year and 10-year survival rates of CC patients. The data from this study may provide valuable information to reduce deaths from GTN.

## Materials and methods

### Selection of patients

We obtained primary data from two databases: 1) SEER database: the SEER * Stat version 8.3.9 was used to capture the required GTN patients (ICD-O-3, C58.9-placenta) between 1975 and 2016; and 2) First Affiliated Hospital of Xi’an Jiaotong University. All patients pathologically diagnosed with GTN at First Affiliated Hospital of Xi’an Jiaotong University between January 2005 and May 2022 were recorded using computerized databases. 

### Processing of data

#### SEER database

**Figure 1.**
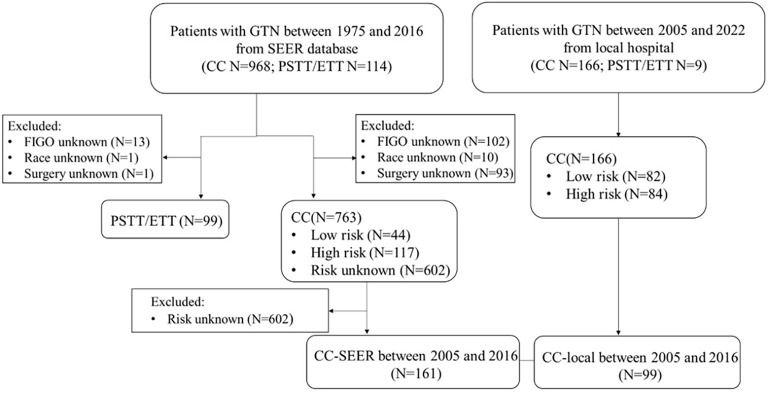
**Flowchart of patient selection from the SEER database and local hospital.** GTN: Gestational trophoblastic neoplasia; CC: Choriocarcinoma; PSTT/ETT: Placental site trophoblastic tumor; SEER: Surveillance, Epidemiology, and End Results.

We collected these data: age at diagnosis, year at diagnosis, race, FIGO stage and prognosis score, marital status (married and unmarried [single, divorced, separated, and widowed]), surgery (including hysterectomy, uterine wedge resection, resection of a solitary pulmonary metastasis or other metastatic site, excluding suction evacuation, curettage, and selective uterine artery embolization), chemotherapy, median household income (< 50% [<US$55.886], ≥ 50% [≥US$55.886]), unemployment (< 50% [<8.48%], ≥ 50% [≥ 8.48%]), for country percentage at least bachelor’s degree (< 50% [29.60%], ≥ 50% [≥29.60%]), county percentage current smokers (age≥18) (< 50% [18.59%], ≥ 50% [≥18.59%]), county percentage current smoker (age≥18) (< 50% [42.97%], ≥ 50% [≥42.97%]), vital status, and survival time. In the SEER database, median household income, education level, unemployment, and smokers are not recorded as individual-level data, so data were analyzed at the county level. Patients for whom the FIGO stage was unknown, as well as race and surgery were excluded. Overall survival (OS) is the time of death from any cause. Finally, 862 patients were eligible for analysis from the first Affiliated Hospital of Xi’an Jiaotong University. The following medical history was reviewed and collected: age at diagnosis, year at diagnosis, chief complaint, previous pregnancy, previous abortion, FIGO stage and prognosis score, surgeries (including total hysterectomy, lesion resection + uterine repair [perforation and bleeding], lesion enucleation [uterine drug resistant], or extrauterine drug-resistant lesion resection [failed to absorb due to multiple chemotherapy], excluding suction evacuation, curettage, and selective uterine artery embolization), chemotherapy, unemployment, bachelor’s degree, body mass index (BMI), and marital status. Finally, 175 patients were eligible for analysis. This study was performed according to the flowchart shown in Figure 1. 

### Ethical statement

Institutional Review Board approval is not required for data used in the SEER database, which is a public research resource. The data used in the First Affiliated Hospital of Xi’an Jiaotong University were approved by the Ethics Committee of the First Affiliated Hospital of Xi’an Jiaotong University (No. XJTU1AF2022LSK-353). 

### Statistical analysis

The clinical and demographic characteristics of the different types of GTN patients (CC and PSTT/ETT), the prognosis score of low-risk, and HR/UHR were compared using the chi-square test (χ^2^) in two data sources. Cox regression analyses were used to identify predictors associated with survival. Kaplan–Meier curves and the log-rank test were performed to evaluate the OS of CC patients. A visualized survival probability was generated using a nomogram, and its accuracy was assessed using the concordance index (C-index). Predicted and actual consistency was assessed by calibration. SPSS (22.0) and R (3.6.3) were used to analyze the data. *P* < 0.05 was considered significant. 

## Results

### Patient characteristics in the database of the local hospital and SEER 

Baseline characteristics of GTN patients stratified by different types are summarized in Tables S1 and S2. At the local hospital, 166 patients were CC and nine were PSTT/ETT patients; in the SEER database, 763 were CC and 99 were PSTT/ETT patients. There was a statistically significant difference between the CC and PSTT/ETT groups in surgery at the local hospital and in age and year of diagnosis in the SEER database. Age under 40 years, low education level, normal BMI, FIGO III/IV stage, high chemotherapy rate, married, three or more pregnancies, two or less miscarriages, and chief complaint of vaginal bleeding constituted the majority of each group in the local hospital. Similarly, age less than 40 years, white race, FIGO III/IV stage, ohigh surgery rate, high chemotherapy rate, and married constituted the majority of each group in the SEER database. At the local hospital, CC patients tended to be under 40 years old, highly unemployed, with normal BMI, married, and had vaginal bleeding as their chief complaint. CC patients tended to be under 40 years old, diagnosed between 1975 and 1998, with FIGO I/II stage, high unemployment rate, and low education level in the SEER database. 

**Table 1:** The comparison of low-risk and HR/UHR patients in local hospital

	**Total (%) (*N* ═ 166)**	**Low risk (%) (*N* ═ 82)**	**HR/UHR (%) (*N* ═ 84)**	***P*-value**
***Age (years)***				
Mean (range)	33.08 (16–55)	32.24 (18–52)	33.93 (16–55)	
<40	126 (75.90)	66 (80.49)	60 (71.43)	0.205
≥40	40 (24.10)	16 (19.51)	24 (28.57)	
***Year of diagnosis***				
2005∼2010	26 (15.66)	17 (20.73)	9 (10.71)	0.122
2011∼2016	73 (43.98)	37 (45.12)	36 (42.86)	
2017∼2022	67 (40.36)	28 (34.15)	39 (46.43)	
***Surgery***				
Yes	73 (43.98)	27 (32.93)	46 (54.76)	**0.005**
No	93 (56.02)	55 (67.07)	38 (45.24)	
***Chemotherapy***				
Yes	158 (95.18)	77 (93.90)	81 (96.43)	0.347
No/Unknown	8 (4.82)	5 (6.10)	3 (3.57)	
***Unemployment***				
No	79 (47.59)	36 (43.90)	43 (51.19)	0.356
Yes	87 (52.41)	46 (56.10)	41 (48.81)	
***Bachelor’s degree***				
Yes	19 (11.45)	10 (12.20)	9 (10.71)	0.811
No	147 (88.55)	72 (87.80)	75 (89.29)	
***BMI*** **(kg/m^2^)**				
<18.5	26 (15.66)	14 (17.07)	12 (14.29)	0.450
18.5–23.9	105 (63.26)	47 (57.32)	58 (69.05)	
24.0–27.9	26 (15.66)	16 (19.51)	10 (11.90)	
≥28	9 (5.42)	5 (6.10)	4 (4.76)	
***FIGO stage***				
I/II	75 (45.18)	52 (63.41)	23 (27.38)	**<0.001**
III/IV	91 (54.82)	30 (36.59)	61 (72.62)	
***Marital status***				
Married	152 (91.57)	75 (91.46)	77 (91.67)	1.000
Unmarried/Divorced/Separated/Single/Widowed	14 (8.43)	7 (8.54)	7 (8.33)	
***Number of pregnancies***				
≤2	64 (38.55)	30 (36.59)	34 (40.48)	0.635
≥3	102 (61.44)	52 (63.41)	50 (59.52)	
***Number of abortions***				
≤2	113 (68.07)	55 (67.07)	58 (69.05)	0.868
≥3	53 (31.93)	27 (32.93)	26 (30.95)	
***Chief complaint***				
Abnormal ultrasonic/β-hCG abnormal	45 (27.11)	27 (32.93)	18 (21.43)	**0.003**
Vaginal bleeding	108 (65.06)	54 (65.85)	54 (64.29)	
Abdominal pain/abdominal distension/other symptoms	13 (7.83)	1 (1.22)	12 (14.28)	

Bolded values are statistically significant (*P* < 0.05). HR/UHR: High/ultra-high risk; BMI: Body mass index; β-hCG: Beta human chorionic gonadotropine.

**Table 2:** The comparison of low-risk and HR/UHR patients in the SEER database

	**Total (%) (*N* ═ 161)**	**Low risk (%) (*N* ═ 44)**	**HR/UHR (%) (*N* ═ 117)**	***P*-value**
***Age (years)***				
Mean (range)	31.60 (16–59)	31.60 (17–53)	31.38 (16–59)	
<40	134 (83.23)	37 (84.09)	97 (82.91)	1.000
≥40	27 (16.77)	7 (15.91)	20 (17.09)	
***Year of diagnosis***				
1999∼2004	6 (3.73)	2 (4.55)	4 (3.42)	0.554
2005∼2010	77 (47.83)	18 (40.91)	59 (50.43)	
2011∼2016	78 (48.44)	24 (54.55)	54 (46.15)	
***Race***				
White	118 (73.29)	33 (75.00)	85 (72.65)	0.702
Black	24 (14.91)	5 (11.36)	19 (16.24)	
Other	19 (11.80)	6 (13.64)	13 (11.11)	
***FIGO stage***				
I/II	32 (19.75)	22 (50.00)	10 (8.55)	**<0.001**
III/IV	129 (80.12)	22 (50.00)	107 (91.45)	
***Surgery***				
Yes	57 (35.40)	25 (56.82)	32 (27.35)	**0.001**
No	104 (64.60)	19 (43.18)	85 (72.65)	
***Chemotherapy***				
Yes	152 (94.41)	40 (90.91)	112 (95.73)	0.258
No/Unknown	9 (5.59)	4 (9.09)	5 (4.27)	
***Marital status***				
Married	91 (56.52)	28 (63.64)	63 (53.85)	0.289
Unmarried/Divorced/Separated/Single/Widowed	70 (43.48)	16 (36.36)	54 (46.15)	
***County-level median household income***				
<50%	85 (52.80)	25 (56.82)	60 (51.28)	0.597
≥50%	76 (47.20)	19 (43.18)	57 (48.72)	
***County percentage unemployed***				
<50%	79 (49.07)	28 (63.64)	51 (43.59)	**0.033**
≥50%	82 (50.93)	16 (36.36)	66 (56.41)	
***County percentage with bachelor’s degree***				
<50%	91 (56.52)	26 (59.09)	65 (55.56)	0.724
≥50%	70 (43.48)	18 (40.91)	52 (44.44)	
***County percentage current smokers (age ≥ 18)***				
<50%	77 (47.83)	22 (50.00)	55 (47.01)	0.860
≥50%	84 (52.17)	22 (50.00)	62 (52.99)	
***County percentage ever smokers (age ≥ 18)***				
<50%	73 (45.34)	19 (43.18)	54 (46.15)	0.289
≥50%	88 (54.66)	25 (56.82)	63 (53.85)	

Bolded values are statistically significant (*P* < 0.05). HR/UHR: High/ultra-high risk; SEER: Surveillance, Epidemiology, and End Results.

### Comparison of low-risk and HR/UHR patients in local hospital and SEER databases

The comparison of low-risk and HR/UHR patients at the local hospital and in the SEER database is shown in Tables 1 and 2. There were 82 low-risk patients and 84 patients with HR/UHR at the local hospital, 44 low-risk patients and 117 patients with HR/UHR in the SEER database. There was a statistically significant difference between the low-risk and HR/UHR groups in the FIGO stage, surgery, and chief complaint in the local hospital and in the FIGO stage, surgery, and unemployment in the SEER database. Age under 40 years, high chemotherapy rate, low education level, normal BMI, married, three or more pregnancies, two or fewer abortions, and chief complaint of vaginal bleeding constituted the majority of each group at the local hospital. Similarly, age less than 40 years, white race, high chemotherapy rate, married, and low income constituted the majority of each group in the SEER database. At the local hospital, HR/UHR patients tended to be 40 years old or older, were diagnosed between 2017 and 2022, had a high surgery rate, FIGO III/IV stage, and complained mainly of abdominal pain, bloating, and other symptoms; while HR/UHR patients from the SEER database were mainly those diagnosed between 2005 and 2015, FIGO III/IV stage, low surgery rate, unmarried, and high unemployment rate. 

### Survival-related factors in the SEER database

In univariate Cox analysis, the following factors were associated with survival: age, race, surgery, marital status, FIGO stage, and unemployment. Multivariate Cox analysis showed that patients older than 40 years (HR 2.094; 95% CI 1.327–3.305) had a worse prognosis than younger patients. In addition, patients with FIGO III/IV stage had worse survival compared with patients with FIGO I/II (HR 1.660; 95% CI 1.082–2.545) (Table 3).

**Table 3:** Univariate and multivariate survival analysis of OS in CC patients in the SEER database

	**Univariable analysis**		**Multivariable analysis**	
**Variable**	**Hazard ratio (95% CI)**	***P-*value**	**Hazard ratio (95% CI)**	***P*-value**
***Age (years)***				
<40	1 (Reference)		1 (Reference)	
≥40	1.942 (1.244–3.033)	**0.003**	2.094 (1.327–3.305)	**0.002**
***Race***				
White	1 (Reference)			
Black	1.718 (1.101–2.680)	**0.017**		
Other	1.019 (0.579–1.794)	0.948		
***Surgery***				
No	1 (Reference)			
Yes	0.601 (0.404–0.893)	**0.012**		
***Chemotherapy***				
No/Unknown	1 (Reference)			
Yes	0.682 (0.415–1.12 2)	0.132		
***Marital status***				
Married	1 (Reference)			
Unmarried/Divorce/Separated/Single/Widowed	1.538 (1.043–2.268)	**0.030**		
***FIGO stage***				
I/II	1 (Reference)		1 (Reference)	
III/IV	1.826 (1.213–2.748)	**0.004**	1.660 (1.082–2.545)	**0.020**
***County-level median household income***				
<50%	1 (Reference)	0.132		
≥50%	1.350 (0.914–1.994)	0.003		
***County percentage unemployed***				
<50%	1 (Reference)			
≥50%	1.559 (1.051–2.31 4)	**<0.027**		
***County percentage with bachelor’s degree***				
<50%	1 (Reference)			
≥50%	1.440 (0.974–2.129)	0.068		
***County percentage current smokers (age ≥ 18)***				
<50%	1 (Reference)			
≥50%	1.170 (0.796–1.720)	0.424		
***County percentage ever smokers (age ≥ 18)***				
<50%	1 (Reference)			
≥50%	1.160 (0.789–1.705)	0.450		

Bolded values are statistically significant (*P* < 0.05). HR/UHR: High/ultra-high risk; SEER: Surveillance, Epidemiology, and End Results; OS: Overall survival.

**Figure 2.**
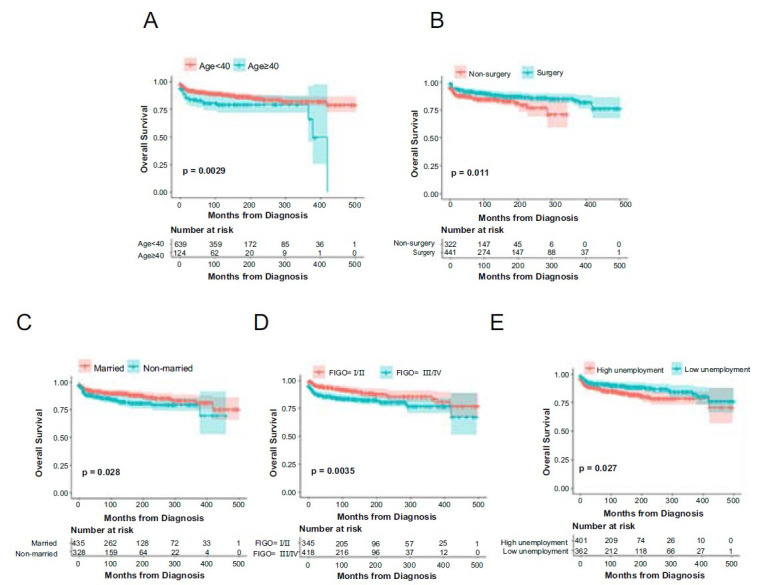
**Kaplan–Meier curves for OS among CC patients: (A) age; (B) surgery; (C) marital status; (D) FIGO stage; (E) unemployment.** OS: Overall survival; CC: Choriocarcinoma.

Kaplan–Meier curves were performed to analyze the effects of prognostic factors on OS of CC patients. OS curves stratified by age are shown in Figure 2A. Significant statistical differences in OS were observed between groups younger than 40 and 40 years or older (10-year OS 88.8% vs 70.3%). OS curves stratified by surgery are shown in Figure 2B. Significant statistical differences in OS occurred between surgery and non-surgery groups (10-year OS 89.2% vs 83.9%). OS curves stratified by marital status are shown in Figure 2C; significant statistical differences in OS were found between married and nonmarried patients (10-year OS 89.4% vs 83.7%). OS curves stratified by the FIGO stage are shown in Figure 2D; significant statistical differences were observed in OS between the FIGO I/II and FIGO III/IV groups (10-year OS 91.2% vs 83.6%). OS curves stratified by unemployment are shown in Figure 2E; significant statistical differences in OS were found between low and high unemployment groups (10-year OS 89.8% vs 84.5%). 

### Construction of a nomogram model and calibration chart for OS of CC patients in the SEER database

**Figure 3.**
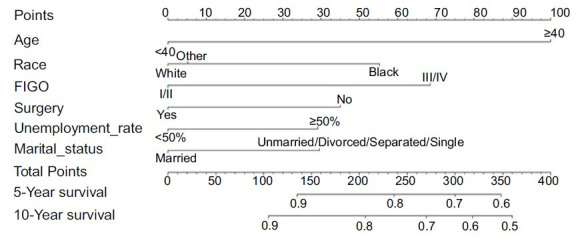
Nomograms to predict 5- and 10-year overall surivival for choriocarcinoma patients.

**Figure 4.**
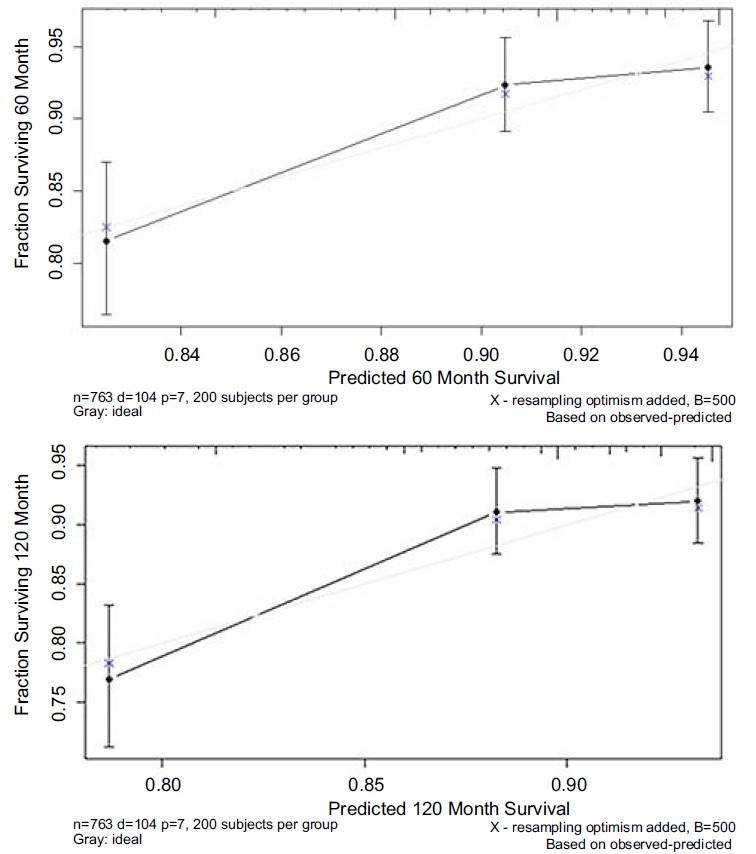
**The calibration plot established for the nomogram among CC patients.** CC: Choriocarcinoma.

A nomogram for the survival of CC patients was created in the SEER database to evaluate 5- and 10-year survival rates (Figure 3). The C-index of the nomogram was 0.674. In addition, calibration charts were created to evaluate the consistency of the nomogram (Figure 4). 

## Discussion

Although the cure rate of GTN is high in developed countries (almost 100%), the mortality rate (20%) is still high in underdeveloped countries [21]. Since most studies on GTN are based on hospital studies with small samples, it is difficult to estimate their incidence. Compared with Western countries, the incidence of GTN is higher in Southeast Asia and Africa [21, 22]. Our hospital is the GTN treatment center in Northwest China. Many cases are referred to our center by secondary hospitals. However, due to non-standard treatment in primary hospitals, there are more cases of chemotherapy resistance. In our previous study, the incidence of invasive mole and CC decreased from 9.0 to 2.7 per 1000 deliveries in 15 years (from 1994 to 2009) [22]. Despite this continuous decrease, the number of these cases was still higher than in some developed countries [21, 22].

GTN has the ability to invade locally and metastasize [10]. The tumor can cause various clinical symptoms, of which vaginal bleeding remains the most common [21]. Our study confirms this observation. In the case of metastatic disease, other symptoms may occur. Lungs are the most common site of metastasis, but it can also spread to the vagina, liver, brain, and intestinal tract [4, 23–25]. Lung lesions may present with symptoms, such as cough and chest pain. Vaginal metastases may be accompanied by vaginal bleeding. Invasion of the central nervous system may cause mild neurological symptoms such as headache [4]. Analyzing data from the local hospital, we found that HR/UHR patients were more likely to have symptoms of metastases. These patients usually visit the physician with some atypical symptoms or signs in addition to ultrasound abnormalities and persistently elevated β-hCG levels or plateaus, which may be the main reason for misdiagnosis and mistreatment.

GTN has a high mortality rate until effective chemotherapy is developed. Studies have shown that GTN had a mortality rate of up to 75% before the advent of chemotherapy [21]. It has been reported that prophylactic administration of either MTX or Act-D chemotherapy during or immediately after molar removal can reduce the incidence of postmolar GTN to 3%–8% [9]. Currently, chemotherapy in combination with surgery improves the prognosis of patients [1]. Although complete remission is achieved in most patients, some of them develop resistance to chemotherapy [1]. Therefore, some researchers recommend that all women with an FIGO risk score of five or six receive first-line therapy with multiple agents, which is much more toxic than single-agent therapy [26]. Floxuridine, Act-D, etoposide, and vincristine (FAEV) and etoposide, MTX, Act-D (EMA)/cyclophosphamide, and vincristine (CO) are commonly used multi-agent chemotherapy regimens for high-risk patients. The main toxicity of FAEV and EMA/CO chemotherapy involves the blood and gastrointestinal tract, with the symptom of grade 4 neutropenia being most prominent with FAEV [27]. Although the medical outcomes of GTN have been extensively studied, we need to explore new influencing factors to more accurately stratify patients for treatment. Previous studies have shown that people with GTN often have a variety of psychological complaints, including anxiety, depression, and reproductive problems [19, 20]. In addition, the emotional burden of patients is further increased by long-term treatment and follow-up. Unfortunately, these symptoms are not taken seriously enough and are not treated promptly. 

The SEER database contains the largest population-based cohort of GTN. In this study, we extracted data on marriage, education, income, unemployment, and smoking habits of patients diagnosed with GTN in the SEER database between 1975 and 2016 to better understand the psychosocial impact of GTN. Our study found marital status had an impact on the prognosis of patients with CC. Married patients had longer survival than unmarried patients, probably because marriage means higher emotional and financial support [28]. As a result, they are more likely to undergo surgery and chemotherapy and thus have access to good health care and hygiene [1, 29]. Other studies have shown that educational level and socioeconomic status are positively correlated with life satisfaction in CC patients [19, 30]. Our study found that employed patients were less likely to get worse. We hypothesize that strong financial power makes it easier for them to access expensive therapies, and therefore their disease is less likely to worsen [31]. In future treatment, we should pay special attention to the unemployed and unmarried group, adjust the treatment plan in time, and strive for more favorable conditions to improve patients’ survival. Previous studies have shown that older age at diagnosis and advanced disease stage are associated with worse outcomes, which was confirmed by our own results. In addition, our data suggest that Black patients have poorer survival compared with non-Black patients [32]. We also constructed a prognostic nomogram. Factors that contributed to high scores included 40 years or older, black race, FIGO III/IV, non-surgery, high unemployment rate, and unmarried family status. Unfortunately, the data of β-hCG and patients’ blood type are not included in the SEER database. Previous studies have shown that blood type A is the most common in GTN patients (49.35%) and the β-hCG level is 50,000–100,000 mIU/mL [33]. Nevertheless, we need to pay attention to the weak associations between factors and patient prognosis caused by selection bias and inadequate control of confounding factors (OR less than 2) [34]. In our future clinical work, we need to verify the authenticity of these conclusions through continuous analysis and practice. 

At the local hospital, our study showed a statistical difference in surgery between CC and PSTT/ETT. It is well known that PSTT/ETT does not respond to MTX and Act-D, so surgery plays a particularly vital role, not only in biopsy of the tissue to confirm the disease but also in prognosis [1, 35]. In the SEER database, we found statistical differences in age and year of diagnosis between CC and PSTT/ETT. The data show that most patients with PSTT/ETT are 40 years old or older and that the number of diagnosed cases increased over time. This is probably related to the slow growth of the tumor and the lack of diagnostic experience in the early years [36]. In addition, we found a statistically significant difference in surgery between low-risk and HR/UHR CC patients. This is because most low-risk patients are cured with monotherapy or hysterectomy, whereas HR/UHR patients often require multiagent chemotherapy, alone or in combination with surgery or radiotherapy [10]. Many patients with HR/UHR metastases require additional surgery even with high-intensity chemotherapy to control bleeding from metastatic site, eliminate chemotherapy resistance, or treat complications during treatment.

To our knowledge, this is the most comprehensive and detailed study of clinical features and demographic characteristics of GTN. The study has some limitations: (1) the SEER database does not provide detailed information about adjuvant therapy, treatment decisions, or pregnancy-related issues; (2) due to the insufficient description of the data in the SEER database, we can only include FIGO III and IV in the same group, which may result in a low OS of FIGO III. In addition, we assume that in the SEER database, the malignancy level of the invasive mole is lower than CC and the prognosis is better, so the corresponding data are not included. Given the rarity of GTN, we focused on CC with many cases for a thorough analysis. Although this study has retrospective limitations, several prognostic factors related to socioeconomic status were included, filling the gaps of previous studies and providing new insights for clinical management of patients.

## Conclusion

In this study, we extracted data on marriage, education, income, unemployment, and smoking habits of patients diagnosed with GTN from the SEER database between 1975 and 2016 to better understand the psychosocial impact of GTN. In conclusion, in addition to some of the currently identified prognostic risk factors, we should improve our understanding of GTN from a socioeconomic perspective and provide effective social support by assessing patients’ clinical and demographic characteristics. Our study might contribute to a rational formulation of the treatment method of GTN and to improve the prognosis of the disease.

## Supplemental data

**Table S1:** Baseline characteristics of eligible GTN patients in local hospital

	**Total (%) (*N* ═ 175)**	**CC (%) (*N* ═ 166)**	**PSTT/ETT (%) (*N* ═ 9)**	***P*-value**
***Age (years)***				
Mean (range)	33.03 (16–55)	33.08 (16–55)	33.23 (21–47)	
<40	134 (76.57)	126 (75.90)	8 (88.89)	0.33
≥40	41 (23.43)	40 (24.10)	1 (11.11)	
***Year of diagnosis***				
2005∼2010	27 (15.43)	26 (15.66)	1 (11.11)	0.342
2011∼2016	75 (42.86)	73 (43.98)	2 (22.22)	
2017∼2022	73 (41.71)	67 (40.36)	6 (66.67)	
***Unemployment***				
No	85 (48.57)	79 (47.59)	6 (66.67)	0.221
Yes	90 (51.43)	87 (52.41)	3 (33.33)	
***Bachelor’s degree***				
Yes	20 (11.43)	19 (11.45)	1 (11.11)	0.632
No	155 (88.57)	147 (88.55)	8 (88.89)	
***BMI *** **(kg/m^2^)**				
<18.5	30 (17.14)	27 (16.27)	3 (33.33)	0.342
18.5–23.9	107 (61.15)	104 (62.65)	3 (33.33)	
24.0–27.9	29 (16.57)	26 (15.66)	3 (33.33)	
≥28	9 (5.14)	9 (5.42)	0 (0.00)	
***FIGO stage***				
I/II	79 (45.14)	75 (45.18)	4 (44.44)	1.000
III/IV	96 (54.86)	91 (54.82)	5 (55.56)	
***Surgery***				
Yes	83 (47.43)	74 (44.58)	9 (100.00)	**0.001**
No	92 (52.57)	92 (55.42)	0 (0.00)	
***Chemotherapy***				
Yes	167 (95.43)	158 (95.18)	9 (100.00)	0.650
No	8 (4.57)	8 (4.82)	0 (0.00)	
***Marital status***				
Married	159 (90.86)	152 (91.57)	7 (77.78)	0.193
Unmarried/Divorced/Separated/Single/Widowed	16 (9.14)	14 (8.43)	2 (22.22)	
***Pregnancy***				
≤2	66 (37.71)	64 (38.55)	2 (22.22)	0.271
≥3	109 (62.29)	102 (61.45)	7 (77.78)	
***Abortion***				
≤2	122 (69.71)	116 (69.88)	6 (66.67)	0.548
≥3	53 (30.29)	50 (30.12)	3 (33.33)	
***Chief complaint***				
Abnormal ultrasonic/hCG abnormal	27 (15.43)	24 (14.46)	3 (33.33)	0.253
Vaginal bleeding	108 (61.71)	104 (62.65)	4 (44.45)	
Abdominal pain/abdominal distension/ other symptoms	40 (22.86)	38 (22.89)	2 (22.22)	

Bolded values are statistically significant (*P* < 0.05). GTN: Gestational trophoblastic neoplasia; CC: Choriocarcinoma; PSTT/ETT: Placental site trophoblastic tumor.

**Table S2:** Baseline characteristics of eligible GTN patients in SEER database

	**Total (%) (*N* ═ 862)**	**CC (%) (*N* ═ 763)**	**PSTT/ETT (%) (*N* ═ 99)**	***P*-value**
***Age (years)***				
Mean (range)	30.75 (14–59)	30.74 (14–59)	31.11 (16–53)	
<40	713 (82.71)	639 (83.75)	74 (74.75)	**0.033**
≥40	149 (17.29)	124 (16.25)	25 (25.25)	
***Year of diagnosis***				
1975∼1998	249 (28.89)	242 (31.72)	7 (7.07)	**<0.001**
1999∼2004	198 (22.97)	173 (22.67)	25 (25.25)	
2005∼2010	222 (25.75)	185 (24.25)	37 (37.37)	
2011∼2016	193 (22.39)	163 (21.36)	30 (30.30)	
***Race***				
White	560 (64.97)	501 (65.66)	59 (59.60)	0.490
Black	168 (19.49)	146 (19.33)	22 (22.22)	
Other	134 (15.54)	116 (15.20)	18 (18.18)	
***FIGO stage***				
I/II	382 (44.32)	345 (45.22)	37 (37.37)	0.162
III/IV	480 (55.68)	418 (54.78)	62 (62.63)	
***Surgery***				
Yes	494 (57.31)	441 (57.80)	53 (53.54)	0.450
No	368 (42.69)	322 (42.20)	46 (46.46)	
***Chemotherapy***				
Yes	726 (84.22)	648 (84.93)	78 (78.79)	0.141
No/Unknown	136 (15.78)	115 (15.07)	21 (21.21)	
***Marital status***				
Married	494 (57.31)	435 (57.01)	59 (59.60)	0.667
Unmarried/Divorced/Separated/Single/Widowed	368 (42.69)	328 (42.99)	40 (40.40)	
***County-level median household income***				
<50%	446 (51.74)	397 (52.03)	49 (49.49)	0.670
≥50%	416 (48.26)	366 (47.97)	50 (50.51)	
***County percentage unemployed***				
<50%	419 (48.61)	362 (47.44)	57 (57.58)	0.069
≥50%	443 (51.39)	401 (52.56)	42 (42.42)	
***County percentage with bachelor’s degree***				
<50%	440 (51.04)	397 (52.03)	43 (43.43)	0.110
≥50%	422 (48.96)	366 (47.97)	56 (56.57)	
***County percentage current smokers (age ≥ 18)***				
<50%	431 (50.00)	375 (49.15)	56 (56.57)	0.200
≥50%	431 (50.00)	388 (50.85)	43 (43.43)	
***County percentage ever smokers (age ≥ 18)***				
<50%	428 (49.65)	377 (49.41)	51 (51.52)	0.749
≥50%	434 (50.35)	386 (50.59)	48 (48.48)	

Bolded values are statistically significant (*P* < 0.05). GTN: Gestational trophoblastic neoplasia; CC: Choriocarcinoma; PSTT/ETT: Placental site trophoblastic tumor.

## References

[1] Borella F, Cosma S, Ferraioli D, Preti M, Gallio N, Valabrega G (2022). From uterus to brain: an update on epidemiology, clinical features, and treatment of brain metastases from gestational trophoblastic neoplasia. Front Oncol.

[2] Hui P (2019). Gestational trophoblastic tumors: a timely review of diagnostic pathology. Arch Pathol Lab Med.

[3] Abu-Rustum NR, Yashar CM, Bean S, Bradley K, Campos SM, Chon HS (2019). Gestational trophoblastic neoplasia, version 2.2019, NCCN clinical practice guidelines in oncology. J Natl Compr Canc Netw.

[4] Lukinovic N, Malovrh EP, Takac I, Sobocan M, Knez J (2022). Advances in diagnostics and management of gestational trophoblastic disease. Radiol Oncol.

[5] Gasparri R, Sedda G, Brambilla D, Girelli L, Diotti C, Spaggiari L (2019). When a differential diagnosis is fundamental: choriocarcinoma mimicking lung carcinoma. J Clin Med.

[6] Li J, Wang Y, Lu B, Lu W, Xie X, Shen Y (2022). Gestational trophoblastic neoplasia with extrauterine metastasis but lacked uterine primary lesions: a single center experience and literature review. BMC Cancer.

[7] Shaaban AM, Rezvani M, Haroun RR, Kennedy AM, Elsayes KM, Olpin JD (2017 Mar-Apr). Gestational trophoblastic disease: clinical and imaging features. Radiographics.

[8] Lok C, Frijstein M, van Trommel N (2021 Jul). Clinical presentation and diagnosis of gestational trophoblastic disease. Best Pract Res Clin Obstet Gynaecol.

[9] Ngan HYS, Seckl MJ, Berkowitz RS, Xiang Y, Golfier F, Sekharan PK (2021 Oct). Diagnosis and management of gestational trophoblastic disease: 2021 update. Int J Gynaecol Obstet.

[10] Soper JT (2021 Feb). Gestational trophoblastic disease: current evaluation and management. Obstet Gynecol.

[11] Winter MC (2021 Jul). Treatment of low-risk gestational trophoblastic neoplasia. Best Pract Res Clin Obstet Gynaecol.

[12] Maestá I, de Freitas Segalla Moreira M, Rezende-Filho J (2020). Outcomes in the management of high-risk gestational trophoblastic neoplasia in trophoblastic disease centers in South America. Int J Gynecol Cancer.

[13] Kong Y, Yang J, Jiang F, Zhao J, Ren T, Li J (2017). Clinical characteristics and prognosis of ultra high-risk gestational trophoblastic neoplasia patients: a retrospective cohort study. Gynecol Oncol.

[14] Strickland AL, Gwin K (2022 May). Gestational trophoblastic disease- rare, sometimes dramatic, and what we know so far. Semin Diagn Pathol.

[15] Silva ALMD, Monteiro KDN, Sun SY, Borbely AU (2021 Dec). Gestational trophoblastic neoplasia: novelties and challenges. Placenta.

[16] Braga A, Mora P, de Melo AC, Nogueira-Rodrigues A, Amim-Junior J, Rezende-Filho J (2019 Feb 24). Challenges in the diagnosis and treatment of gestational trophoblastic neoplasia worldwide. World J Clin Oncol.

[17] Hancock BW, Tidy J (2021 Jul). Placental site trophoblastic tumour and epithelioid trophoblastic tumour. Best Pract Res Clin Obstet Gynaecol.

[18] Ngu SF, Ngan HYS (2021 Jul). Surgery including fertility-sparing treatment of GTD. Best Pract Res Clin Obstet Gynaecol.

[19] Di Mattei V, Mazzetti M, Perego G, Rottoli S, Mangili G, Bergamini A (2021). Psychological aspects and fertility issues of GTD. Best Pract Res Clin Obstet Gynaecol.

[20] Blok LJ, Frijstein MM, Eysbouts YK, Custers J, Sweep F, Lok C (2022). The psychological impact of gestational trophoblastic disease: a prospective observational multicentre cohort study. BJOG.

[21] Freitas F, Braga A, Viggiano M, Velarde LGC, Maesta I, Uberti E (2020). Gestational trophoblastic neoplasia lethality among Brazilian women: a retrospective national cohort study. Gynecol Oncol.

[22] Sun R, Zhang Y, Zheng W, Tian Q, An R, Xue Y (2016). Clinical characteristics of gestational trophoblastic neoplasia: a 15-year hospital-based study. Int J Gynecol Cancer.

[23] Chen F, Tatsumi A, Numoto S (2001). Combined choriocarcinoma and adenocarcinoma of the lung occurring in a man: case report and review of the literature. Cancer.

[24] Wang Y, Wang Z, Zhu X, Wan Q, Han P, Ying J (2022). Intestinal metastasis from choriocarcinoma: a case series and literature review. World J Surg Oncol.

[25] Wei H, Zhang T, Liu B, Xue X, Wang G (2016). Choriocarcinoma of unknown origin with multiple organ metastasis and cerebral hemorrhage: a case report and literature review. Oncol Lett.

[26] Braga A, Paiva G, Ghorani E, Freitas F, Velarde LGC, Kaur B (2021 Aug). Predictors for single-agent resistance in FIGO score 5 or 6 gestational trophoblastic neoplasia: a multicentre, retrospective, cohort study. Lancet Oncol.

[27] Ji M, Jiang S, Zhao J, Wan X, Feng F, Ren T (2022). Efficacies of FAEV and EMA/CO regimens as primary treatment for gestational trophoblastic neoplasia. Br J Cancer.

[28] Zhang SL, Wang WR, Liu ZJ, Wang ZM (2019). Marital status and survival in patients with soft tissue sarcoma: a population-based, propensity-matched study. Cancer Med.

[29] Uchino BN (2006). Social support and health: a review of physiological processes potentially underlying links to disease outcomes. J Behav Med.

[30] Cagayan MSFS, Llarena RT (2010). Quality of life of gestational trophoblastic neoplasia survivors: a study of patients at the Philippine General Hospital trophoblastic disease section. J Reprod Med.

[31] Xie JC, Yang S, Liu XY, Zhao YX (2018). Effect of marital status on survival in glioblastoma multiforme by demographics, education, economic factors, and insurance status. Cancer Med.

[32] Tarney CM, Tian C, Craig ER, Crothers BA, Chan JK, Gist GD (2018). Relative effects of age, race, and stage on mortality in gestational choriocarcinoma. Int J Gynecol Cancer.

[33] Jagtap SV, Aher V, Gadhiya S, Jagtap SS (2017). Gestational trophoblastic disease - clinicopathological study at tertiary care hospital. J Clin Diagn Res.

[34] Grimes DA, Schulz KF (2012). False alarms and pseudo-epidemics: the limitations of observational epidemiology. Obstet Gynecol.

[35] Sebire NJ, Lindsay I (2010). Current issues in the histopathology of gestational trophoblastic tumors. Fetal Pediatr Pathol.

[36] Frijstein MM, Lok CAR, van Trommel NE, Ten Kate-Booij MJ, Massuger LFAG, van Werkhoven E (2019 Feb). Management and prognostic factors of epithelioid trophoblastic tumors: results from the international society for the study of trophoblastic diseases database. Gynecol Oncol.

